# Complete Genome Sequence of *emm* Type 14 *Streptococcus pyogenes* Strain HSC5

**DOI:** 10.1128/genomeA.00612-13

**Published:** 2013-08-15

**Authors:** Gary C. Port, Elyse Paluscio, Michael G. Caparon

**Affiliations:** Department of Molecular Microbiology, Washington University School of Medicine, St. Louis, Missouri, USA

## Abstract

*Streptococcus pyogenes* causes a greater diversity of human disease than any other bacterial pathogen. Here, we present the complete genome sequence of the *emm* type 14 *S. pyogenes* strain HSC5. This strain is a robust producer of the cysteine protease SpeB and is capable of producing infection in several different animal models.

## GENOME ANNOUNCEMENT

*Streptococcus pyogenes* is an important Gram-positive human pathogen capable of causing a wide range of diseases, ranging from mild superficial skin and throat infections (impetigo and “strep throat”) to more-severe invasive diseases, including cellulitis and necrotizing fasciitis, as well as numerous postinfection sequelae, including acute rheumatic fever and poststreptococcal glomerulonephritis (1). As a strict human pathogen with no environmental or animal reservoirs, there is no single animal model that entirely replicates the diversity of human diseases caused by *S. pyogenes*. Consequently, several different animal models have been developed that mimic particular aspects of *S. pyogenes* pathogenesis. The *emm* type 14 *S. pyogenes* strain HSC5 (2) is capable of causing robust and highly reproducible infections in multiple animal models, including a necrotizing fasciitis (myositis) model of infection in zebrafish (*Danio rerio*) (3) and several distinct mouse models, including a self-resolving subcutaneous ulcer model (4), a lethal systemic infection following intraperitoneal injection, and a recently described asymptomatic murine mucosal carriage model (5).

One of the most well-studied secreted virulence factors produced by *S. pyogenes* is the cysteine protease SpeB, which is abundantly produced in many strains and is subject to multiple layers of regulation (6). HSC5 has proven to be an exquisitely sensitive SpeB indicator strain, as mutagenesis experiments in HSC5 have revealed several novel SpeB regulators (7–10). Furthermore, studies on HSC5 have provided numerous insights into the fundamental aspects of protein secretion in *S. pyogenes*, including the role of the signal recognition particle (SRP) pathway (11) and the ExPortal protein secretion microdomain (12). We therefore sought to determine the complete genome sequence of HSC5, the first *emm* type 14 strain to be sequenced, in order to provide a framework for future genetic studies on this versatile strain.

Genomic DNA from HSC5 was purified by phenol-chloroform extraction (13) and sequenced using a 454 GS FLX sequencer (MOgene LC, St. Louis, MO) by collecting shotgun reads and 8-kb paired-end reads. A total of 202,164 reads (64,290,146 nucleotides) were generated, reaching a depth of 35-fold genome coverage. Sequences were assembled using Newbler v2.5.3. A total of 26 contigs were assembled and aligned to the *S. pyogenes* strain SF370 genome, generating a single scaffold that was 96% complete. The 26 remaining gaps (ranging from 0.5 kb to 16 kb, for a total of 70 kb) were filled in by primer walking (IDT, Coralville, IA) and Sanger sequencing (GENEWIZ, South Plainfield, NJ). To correct any sequencing errors, the genomic DNA was resequenced by Illumina HiSeq 2000 (Genome Technology Access Center [GTAC], Washington University, St. Louis, MO) by collecting 50-bp single-end reads, generating a total of 9,574,454 reads (403,593,696 nucleotides), reaching a depth of 222-fold genome coverage. Illumina data were aligned to the reference HSC5 scaffold sequence using DNAStar SeqMan NGen 4.0.0 (DNAStar) to generate a final consensus sequence. The HSC5 genome is composed of 1,818,351 bp, with an average G+C content of 38.5%.

### Nucleotide sequence accession number.

The complete whole-genome sequence of *S. pyogenes* strain HSC5 has been deposited in the NCBI under the accession no. CP006366.
